# Are 'Village Doctors' in Bangladesh a curse or a blessing?

**DOI:** 10.1186/1472-698X-10-18

**Published:** 2010-07-06

**Authors:** Shehrin S Mahmood, Mohammad Iqbal, S M A Hanifi, Tania Wahed, Abbas Bhuiya

**Affiliations:** 1 Social and Behavioural Sciences Unit, Public Health Sciences Division, ICDDR, B GPO Box 128, Dhaka 1000 Bangladesh

## Abstract

**Background:**

Bangladesh is one of the health workforce crisis countries in the world. In the face of an acute shortage of trained professionals, ensuring healthcare for a population of 150 million remains a major challenge for the nation. To understand the issues related to shortage of health workforce and healthcare provision, this paper investigates the role of various healthcare providers in provision of health services in Chakaria, a remote rural area in Bangladesh.

**Methods:**

Data were collected through a survey carried out during February 2007 among 1,000 randomly selected households from 8 unions of Chakaria *Upazila*. Information on health-seeking behaviour was collected from 1 randomly chosen member of a household from those who fell sick during 14 days preceding the survey.

**Results:**

Around 44% of the villagers suffered from an illness during 14 days preceding the survey and of them 47% sought treatment for their ailment. 65% patients consulted Village Doctors and for 67% patients Village Doctors were the first line of care. Consultation with MBBS doctors was low at 14%. Given the morbidity level observed during the survey it was calculated that 250 physicians would be needed in Chakaria if the patients were to be attended by a qualified physician.

**Conclusions:**

With the current shortage of physicians and level of production in the country it was asserted that it is very unlikely for Bangladesh to have adequate number of physicians in the near future. Thus, making use of existing healthcare providers, such as Village Doctors, could be considered a realistic option in dealing with the prevailing crisis.

## Background

Bangladesh has an acute shortage of health workforce to provide health services to its 150 million people [1]. A nationwide survey in 2007 estimated that there is a shortage of 60,000 physicians, 280,000 nurses and 483,000 technologists in the country. With the current level of production, it is very unlikely that the nation will recover this shortage in near future [2]. The current composition of the health workforce in the country is dominated by informal providers, constituting 95% of total workforce and the share of the trained professionals is low at 5% [2]. The number of informal healthcare providers, especially the Village Doctors, has increased phenomenally during the last decade which clearly indicates an increasing market demand. With ongoing emphasis on expansion of the public-sector health services, manned by formally trained providers, it is important to know how meaningful it is given the reality in the community [3]. It is against this background that this paper is written with an aim to assess the role of various healthcare providers in the present day service provision and discuss realistic options in ensuring health for the rural masses.

## Methods

Data were collected from the villagers through a survey. Informed consent was taken verbally from the respondents and they had complete right to withdraw from providing information at any point of the interview. The study was reviewed by the Ethical Review Committee of the International Centre for Diarrhoeal Disease Research, Bangladesh (ICDDR, B) which follows the international standard for research ethics. Detailed methodology of the study is presented below.

### Study area and study period

The present study was carried out in Chakaria, a remote rural area in Bangladesh. Administratively, it is under Cox's Bazar district. The population density is 782 individuals per square kilometre. The highway from Chittagong to Cox's Bazar passes through Chakaria. The east side of Chakaria is hilly, while on the west side towards the Bay of Bengal, is lowland.

The health and other development indicators of the area lag behind those for the rest of the country. The total fertility rate in Chakaria during 2007 was 3.5. The infant and under-five mortality rates during 2007 were 48.0 and 63.4 respectively. Life expectancy at birth was 67.2 years for males and 69.7 years for females in 2007 [4]. However, the challenges facing the health systems of the area are common to those facing the rest of rural Bangladesh and therefore the conclusions derived from this area can be generalized for the rest of the country.

The survey was carried out during February 2007.

### Data collection

Data presented in this paper come from eight of the 18 Unions of Chakaria *Upazila *(sub-district). A Health and Demographic Surveillance Systems (HDSS), known as the Chakaria HDSS, runs in these eight unions which gives information on the demographic, socioeconomic and other health indicators of the area on a quarterly basis. Chakaria is an INDEPTH member site [5]. It collects data from 7,600 systematically randomly-chosen households out of 26,979 households of the eight unions of Chakaria.

The current survey was carried out among 1,000 households randomly selected from the 7,600 households of Chakaria HDSS. Information on illness prevalence was collected from a total of 6,162 members who were living in these 1,000 households at the time of data collection.

From these selected households 892 households had at least one member who was sick during the 14 days preceding data collection. For the households that had more than one sick member, one was selected randomly for data on health seeking pattern. Among these 892 household members, 120 were not available at the time of data collection, 2 refused to participate in the survey and there were missing information on another 5 members. Therefore, information on health seeking pattern was collected from 765 household members who were sick during the 14 days preceding the survey.

### Instruments

A structured questionnaire was developed in *Bangla *to collect information on health-seeking behaviour of the villagers. The questionnaire was administered on villagers who have been sick during the 14 days preceding the survey. This questionnaire was pre-tested outside the study area in order to ensure consistency, appropriateness of language and sequencing of the questions. Based on the feedback from pre-testing, the questionnaire was modified and rephrased where necessary. Data were collected on: type and symptoms of illnesses, care-seeking behaviour during illness including home remedy and consultation with healthcare providers, type of health care provider contacted, and the socioeconomic characteristics of the households. Information on type and symptoms of illness were collected through open-ended questions.

### Respondents

Respondents were the adult sick persons themselves. For the sick children the respondent was either their mother or their caregiver. In total, health-seeking behaviour was recorded for 765 individuals.

Among the sick household members 36% were male and 64% were female and their mean age was 26 years varying from newborn to 93 years of age. Majority of these household members (51%) aged over five years were illiterate, 29.5% had one to five years of education and 19.5% had six or more years of education with the maximum not exceeding 14 years of education.

### Interviewers

21 interviewers with 12 years or more of formal education were recruited for data collection, majority of whom had previous field experience. Two supervisors supervised the interviewers in two groups. Interviewers received in-class training for 3 days and had field practice for another 2 days followed by a long de-briefing by the supervisors at the end of each day. An instruction manual explaining the key terms in the questionnaire was developed and provided to the interviewers as a guide.

### Data management and analysis

Each questionnaire was scrutinized in the field and at the field office on the same day of the interview. The supervisors besides the day-to-day supervision, re-interviewed 3% of the households and any inconsistencies identified between the two interviews were corrected. Inconsistencies identified while checking the questionnaires were sorted out through additional field visits, if needed, by the supervisors. Data entry using Foxpro database software started within two days of the start of data collection.

Data analysis was carried out using SPSS. Cross tabular analysis was carried out. Chi-square test has been used to test the statistical significance of relationships.

The socioeconomic status used to analyse the data was derived using asset index. The list of assets is similar to the one that is in use at the Chakaria HDSS which included Van/rickshaw, bicycle, motorcycle, television, telephone/cell phone, radio, watch/clock, couch, chair, table, bed (khat/chouki), mosquito net, quilt/blanket, electricity connection, sewing machine, tube well, sanitary latrine, mattress, and *Almira *(closet/cabinet) [4]. All these assets were coded as dichotomous variables where ownership of an asset was coded as 1 or 0 otherwise. Each household asset for which information was collected was assigned a weight equal to the factor score generated through a principal components analysis. We followed the SPSS factor analysis procedure. Individuals were then ranked according to the total score of the households they belong to [6-8]. Finally using these scores the sample was stratified in five quintiles from lowest to the highest quintile; the higher the quintiles, the better-off the households.

### Variables

#### Health-seeking behaviour

Health-seeking behaviour has been defined as a "sequence of remedial actions that individuals undertake to rectify perceived ill-health"[9-11].

#### Illnesses

The illnesses mentioned in this report are based on reported symptoms or names of illness reported by the respondents themselves. In case of mentioning a name of an illness symptoms were also recorded. For ease of analysis, diseases of similar type were grouped together based on the coding scheme developed by a physician. The broad categories in which diseases were grouped together were: respiratory tract diseases, infectious diseases, neurological diseases, gastro-intestinal tract diseases, skin and soft tissue diseases, musculo-skeletal diseases, kidney and urinary tract diseases, eye problem, cardiovascular diseases, cancer, cold/fever, diarrhoeal diseases, and hepatobilliary. If a single patient mentioned multiple illnesses within the reporting time period, they were recorded. However, for our current analysis health-seeking behaviour for that particular patient was recorded for the most recent illness.

#### Home remedy

Any remedial action undertaken by an individual without consulting a healthcare provider has been considered as 'home remedy'.

#### Village Doctor

For the current analysis Village Doctors were defined as informal healthcare providers and or drug vendors practicing allopathic medicine. Village Doctors practicing homeopath or other traditional medicines were not included in the analysis.

In 2007, a survey was carried out to investigate the characteristics of Village Doctors practicing allopathic medicine in Chakaria. It was found that of the total 328 Village Doctors practicing allopathic medicine 95% were male and the mean age of Village Doctors was 39 years. All the Village Doctors had passed at least 7^th ^grade of education. However, only 4% had government accredited training in the system of medicine that they were practicing whereas others had non-accredited trainings of various durations. Village Doctors had embarked on the profession by attending courses or trainings, by being a trainee in a drug store, assistant in a doctor's chamber or of a Village Doctor or by inheriting the livelihood from a family member. One important fact was that majority (82%) of the Village Doctors in Chakaria sell drugs alongside their practice [12].

#### Socioeconomic status

The socioeconomic status of the individuals was derived by using the asset quintile approach and the individuals were grouped into five quintiles: lowest quintile (poor), second quintile, third quintile, fourth quintile and the highest quintile (better-off).

## Results

### Illness Pattern

In Chakaria, 43.5% of the 6,162 individuals included in the survey reported suffering from some kind of illness during the 14 days preceding the survey. Around 41% of these patients were still sick on the day of the interview.

Among the reported illness cold and fever occupied the major share and 52.2% of the patients reported suffering from cold and fever during the 14 days preceding the survey. A wave of viral fever persisting during the data collection period could have been responsible for such high rates of illness. Among the other reported illnesses, musculo-skeletal diseases (9.7%) including aches in different parts of the body, and gastro-intestinal diseases (9.7%) were the second most prevalent category. Another 6.1% of the patients suffered from neurological diseases and 5% suffered from Respiratory tract diseases. Diarrhoeal disease was reported by 4.8% of the patients. The pattern of reported illness is presented in table 1.

**Table 1:** Pattern of reported illness

Diseases	% of responses (n)
Cold/Fever	52.2 (399)

Musculo-skeletal diseases	9.7 (74)

Gastro-intestinal tract diseases	9.7 (74)

Neurological disease	6.1 (47)

Respiratory tract diseases	5 (38)

Diarrhoeal diseases	4.8 (37)

Skin and soft tissue diseases	3.1 (24)

Cardiovascular diseases	3 (23)

Infectious diseases	2.2 (17)

Eye problem	1.7 (13)

Kidney and urinary tract diseases	0.4 (3)

Hepatobilliary	0.4 (3)

Cancer	0.1 (1)

Other	1.6 (12)

Total	100 (765)

### Treatment seeking

Forty-seven percent of the 765 randomly chosen patients sought treatment for their illness from a healthcare provider. Information on reasons behind not seeking care was collected from those who did not seek care. Of the 407 household members who did not seek care for their illness, 51% (206) did not feel that the disease required any treatment and 1.5% (6) got cured without any treatment. Around 3% (11) were waiting for the disease to get serious enough to consult a healthcare provider. Five percent (20) patients were self-medicating themselves based on a past prescription of some health care provider for similar health problems, whereas, 2% (8) depended on self-medication without previous prescription. Forty percent (161) did not have enough money to consult a healthcare provider. Only 1.7% (7) mentioned that there were no healthcare providers available nearby. Some patients also mentioned that they could not make the time to seek treatment while others were neither in a state to contact the healthcare provider, nor could they avail treatment through someone else. Around 2% (8) did not seek treatment, as they did not believe that treatment would make a difference.

### Source of treatment: Public Vs. Private and Qualified Vs. Non-qualified care

Patients sought treatment from both public and private health facilities. However, utilization of public health centres was found to be very low (4.7%) compared to that of the private facilities (68.4%). Another 16.4% went to the pharmacies or drug stores and for 6.4% of the patients the providers made house calls. NGO facilities were availed by 1.8% and 0.6% patients mentioned consulting healthcare providers over the phone.

**Figure 1:**
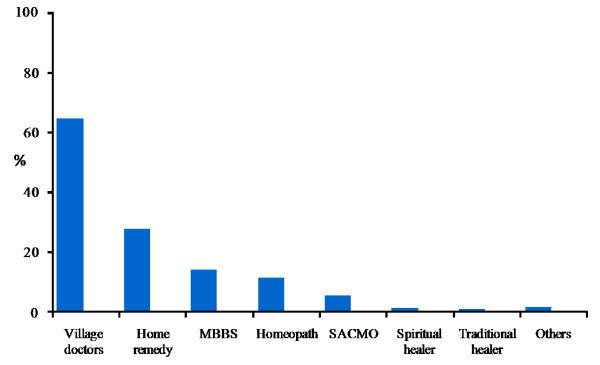
**Type of healthcare provider consulted**. Note: Multiple responses recorded. SACMO = Sub-assistant community medical officer.

Regarding the type of treatment, results show that around 65% of the patients consulted Village Doctors at some point of treatment and for 46% of the patients Village Doctors were the sole source of care for that particular illness. On the other hand, consultation with an MBBS doctor in combination with other types of healthcare providers was as low as 14.2%. Proportion of patients consulting only MBBS doctors for the whole duration of illness was even lower (9.4%). Around 12% sought homeopathic treatment at some stage of their illness, 2.5% depended on spiritual/traditional healers and 5.6% depended solely on home remedy (figure 1).

The findings further indicate that villagers prefer to consult Village Doctors in treating almost all types of illnesses. Looking at the treatment-seeking pattern for the most frequently reported illnesses Village Doctors were found to be the most popular source of healthcare (table 2). They were also the first line of care or first choice of treatment for 66.7% of the patients. In contrast, MBBS doctors with formal qualification were the first line of care for only 12.1% of the patients. For another 12.1% homeopathic doctors were the first choice. Few people also consulted paramedics or traditional healers on their first visit.

**Table 2:** Disease specific preference for various healthcare providers

Disease (n = 360)	Type of health care provider	% (n)
Cold/Fever	Village Doctors	77.0 (194)
	
	Home remedy	24.6 (62)
	
	Homeopathic doctors	8.7 (22)
	
	MBBS	8.3 (21)
	
	Paramedics/SACMO	5.2 (13)
	
	*Kabiraj*(traditional healer)	0.4 (1)
	
	*Pir/Fakir*(spiritual healer)	0 (0)

Musculo-skeletal diseases	Home remedy	56.7 (17)
	
	Village Doctors	53.3 (16)
	
	MBBS	16.7 (5)
	
	Homeopathic doctors	13.3 (4)
	
	Paramedics/SACMO	3.3 (1)
	
	*Pir/Fakir *(spiritual healer)	3.3 (1)
	
	*Kabiraj *(traditional healer)	0 (0)

Gastro intestinal diseases	Village Doctors	63.3 (19)
	
	Homeopathic doctors	20 (6)
	
	MBBS	16.7 (5)
	
	Home remedy	10 (3)
	
	Paramedics/SACMO	3.3 (1)
	
	*Kabiraj *(traditional healer)	3.3 (1)
	
	*Pir/Fakir*(spiritual healer)	3.3 (1)

Neurological diseases	Village Doctors	68.8 (11)
	
	Home remedy	31.3 (5)
	
	MBBS	25 (4)
	
	Paramedics/SACMO	6.3 (1)
	
	*Pir/Fakir*(spiritual healer)	6.3 (1)
	
	Homeopathic doctors	0 (0)
	
	*Kabiraj*(traditional healer)	0 (0)

Respiratory tract diseases	Village Doctors	59.3 (16)
	
	Home remedy	29.6 (8)
	
	MBBS	25.9 (7)
	
	Homeopathic doctors	7.4 (2)
	
	Paramedics/SACMO	7.4 (2)
	
	*Pir/Fakir *(spiritual healer)	3.7 (1)
	
	*Kabiraj*(traditional healer)	0 (0)

Diarrhoeal diseases	Home remedy	57.1 (12)
	
	Village Doctors	47.6 (10)
	
	MBBS	14.3 (3)
	
	Homeopathic doctors	4.8 (1)
	
	Paramedics/SACMO	4.8 (1)
	
	*Kabiraj *(traditional healer)	0 (0)
	
	*Pir/Fakir*(spiritual healer)	0 (0)

Note: multiple responses recorded

Most of the patients did not feel the need to consult a second healthcare provider. Only 30 patients went on for further consultation. Almost half of these patients (14) went on to consult a Village Doctor, which again supports their popularity in the area. However, it should be noted that a large share (13) of those who opted for a consultation with a second healthcare provider had their initial consultation with a Village Doctor.

Most of the patients (73%) chose their healthcare provider based on the belief that they were receiving quality healthcare from them. Around 38% of the patients reported that they chose the healthcare provider as they were the closest to their homes. The cost of treatment was another important reason for choosing healthcare providers. Many patients preferred health care providers who offer low-cost treatment or provide treatment on credit. The behaviour of the healthcare provider (14%), prior acquaintance with the healthcare provider (4%) and the lack of choice among healthcare providers practicing nearby (4.7%) were mentioned as reasons for choosing a particular healthcare provider.

### Socio-economic status and health-seeking behaviour

The health-seeking behaviour of the villagers in Chakaria was analyzed separately for patients from five different socioeconomic statuses: lowest quintile (Poor), second quintile, third quintile, fourth quintile and highest quintile (better-off).

### Illness reporting and treatment seeking in the various asset quintiles

A significantly higher proportion of patients from the lowest quintile (47.3%) reported being ill during the 14 days preceding the survey compared to those in the highest quintile (42%). As reported earlier treatment seeking behaviour was recorded for one randomly chosen patient per household and despite the higher reporting of illness in the lowest quintile, treatment seeking did not vary across the socioeconomic quintiles (table 3).

**Table 3:** Illness and contact with healthcare provider by household asset quintiles

Asset quintile	Number of respondents	% sick during 14 days preceding the survey	P value	No of sick individuals interviewed	% contacted a healthcare provider (n)	P value
Highest (Better off)	1375	42.0	0.002	156	53.2 (83)	0.095
			
4^th^	1372	40.5		152	40.8 (62)	
			
3^rd^	1240	42.7		160	52.5 (84)	
			
2^nd^	1090	46.2		138	44.9 (62)	
			
Lowest (poor)	1085	47.3		160	43.1 (69)	
			
Total	6162	43.5		766	47.0 (360)	

**Figure 2:**
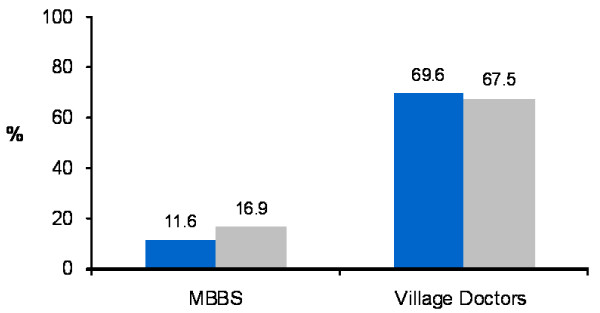
**Choice of healthcare provider among patients from lowest and highest quintiles**. Blue bar = Lowest quintile Gray bar = Highest quintile.

Among the patients who consulted a health care provider, 11.6% patients in the lowest quintile went to MBBS doctors compared to 16.9% in the highest quintile. Seeking care from Village Doctors was more among patients from the lowest quintile (69.6%) compared to the patients from the highest quintile (67.5%) (figure 2). Although these differences were not statistically significant, an overall increasing trend in consulting MBBS doctors among patients from the higher socioeconomic quintiles was observed. One interesting finding is that Village Doctors are popular among patients from all socioeconomic status.

## Discussion

This paper focuses on the role of various healthcare providers, especially the Village Doctors in delivering healthcare services in Chakaria, a remote rural area in Bangladesh. Data showed that 43.5% of the people in Chakaria suffered from illness during a 14-day time period and among them less than half (47%) sought treatment. Findings further indicate that healthcare need in Chakaria in mostly met by the not-so-qualified Village Doctors. The Village Doctors, therefore, are identified as the key actors in the health systems of the area providing 65% of healthcare irrespective of type of illnesses. In contrast, use of trained healthcare professionals, particularly MBBS doctors, is low at only 14%. Earlier studies have also identified the informal healthcare providers, including Village Doctors, as the major source of healthcare in rural Bangladesh [13-16]. Results from the study also show Village Doctors to be the first choice of treatment in most cases and the only choice in some cases for the community people.

The socioeconomic status (SES) of individuals is believed to influence their health-seeking behaviour [15,17-19]. The current study analyzed the relationship between individual's socioeconomic status and their choice of healthcare provider. Looking into the health-seeking pattern among patients from various asset quintiles in Chakaria we found that consultation with Village Doctors did not vary significantly with socioeconomic status. This indicates the popularity of Village Doctors among patients from all socioeconomic status.

A study carried out in 2007 evaluated the practice of the Village Doctors in Chakaria and showed that these Village Doctors resort to inappropriate and even harmful practices [12]. Other studies have also questioned the quality of services and the level of knowledge of the Village Doctors practicing in rural Bangladesh [17,20-22]. Despite this, in the current study villagers expressed that they consulted Village Doctors based on the belief that they are receiving quality health care. This reflects the existence of a strong social trust between the villagers and the Village Doctors, which eventually makes them the dominant source of healthcare in the area. This satisfaction with Village Doctors and the causes behind have been documented in earlier studies as well [15]. Findings from a qualitative study carried out in 2007 to investigate the Village Doctors and their patients in Chakaria showed that issues like round-the-clock availability, provision of house calls, lower consultancy fee, referral linkage, one-stop service for treatment and medicine, and availability of medicines according to ability-to-pay, make Village Doctors the most preferred healthcare provider [12].

The low level of utilization of the MBBS doctors in Chakaria, which is typical of the rest of the rural areas of the country, is of concern [15]. A study conducted on the health workers (i.e. doctors, nurses, and midwives) in Chakaria showed that there are only 72 health workers in the area [12]. With a population of 503,390, the health worker density in Chakaria is only 0.14/1,000 population which falls far short of the WHO recommended health worker density of 2.5/1,000 population that is required to achieve the Millennium Development Goals [1,23]. This means that the health systems of Chakaria is operating with a shortage of 1,258 health workers. The finding of this study also reflects on the shortage of health manpower in Chakaria. With a 44% illness prevalence rate in a 14 day time period, Chakaria can be expected to have approximately 16,000 people suffering from some kind of illness on a single day. According to the present study around 47% of the patients sought healthcare. Applying the current treatment seeking rate, 7,500 patients can be expected to demand healthcare on a single day in Chakaria. If for instance, all these estimated 7,500 patients decide to consult a qualified healthcare provider, specifically an MBBS, on a single day, then the existing pool of physicians (i.e. 39 MBBS doctors) within Chakaria will not suffice. This would mean a provider would then have to treat around 190 patients per day to ensure universal coverage, which is quite unrealistic. Assuming a patient load of 30 per day it would require about 250 physicians to fulfill this gap in demand. However, the limitation of our finding is that the high rate of viral fever observed during the study period inflated the prevalence rate of illness and thus our estimation of required number of physicians. If we base our estimation on a lower prevalence rate of 30% and keep all other assumptions constant, the required number of physicians would still be 170.

Therefore, regardless of the illness rate, the size of demand for healthcare in Chakaria definitely indicates towards an expansion of the existing health workforce. However, one should bear in mind that even if actions to expand the health workforce are taken now, effects will only begin to be felt years later as the training process of producing physicians and other health workers is lengthy and the training institutes are not sufficient to meet this demand fast enough. The current turn out rate of the medical institutes in the country is around 5,000 physicians per year and the distribution of the physicians is heavily concentrated in the urban areas. In addition, many of these newly graduated qualified physicians migrate to jobs abroad. Hence, waiting for enough new workers to graduate through the conventional system and to engage in healthcare provision of the rural areas will mean lengthy delays in providing urgently needed services. Thus, measures to raise recruitment rates and expand training facilities, although important, are not the whole solution. On the other hand, diverting resources towards expanding the pool of physicians would not necessarily result in increased utilization of services of qualified physicians. Factors like cost of treatment, travelling distance, behaviour towards patients, distributional bias between the rural and urban areas, would still control the access and availability of their services [13]. Therefore, in addition to these measures, alternative and simplified models need to be developed that can quickly expand the capacity of the current health workforce. Innovative methods, like task shifting can shorten this delay effect [24,25]. Given the fact that not all cases need to be attended by a physician, task shifting can help us make efficient use of the human resources that are currently available in the locality [25].

In this line of thought, making use of the available pool of under-skilled providers, particularly the Village Doctors, can be a feasible option. As mentioned earlier, currently there exists a vast army of Village Doctors in Chakaria and they are the dominant source of healthcare for the community people. These Village Doctors, if trained in proper management of common ailments, can be a potential source of quality healthcare for the villagers. This can increase the pool of human resources for health very rapidly and can serve the ultimate goal of ensuring quality healthcare for all. Studies from Vietnam, Laos, Thailand and Nepal have shown that it is possible to improve the knowledge and practice of these semi or unqualified providers regarding rational use of drugs including prevention of misuse of antibiotics through proper training [26-28]. In 1978 Bangladesh government trained 16,000 informal healthcare providers, called "*Palli Chikitshaks*" or "Village Doctors". This was similar to the concept of "barefoot doctors" in China [29,30]. Unfortunately, this programme was later abandoned due to resource constraint and regulatory issues. These trained Village Doctors still practice in the rural areas and their services are considered better compared to the other informal providers in terms of quality [3]. On the other hand, informal healthcare providers have successfully been used to deliver DOTS services in managing tuberculosis in Bangladesh [31]. So far, the major challenges facing these models of utilizing community based agents to provide health services in the country lie with issues like competence, trust and sustainability of the programmes [32]. The Village Doctors could also contribute in strengthening the health systems of the country by making proper and timely referrals to qualified physicians. However, this warrants building a productive network where the services of Village Doctors and the physicians would complement each other. It should be mentioned that there exists mixed reaction to the idea of training community based healthcare providers, i.e., Village Doctors, from the concerned medical associations of the country. The executive members of the Bangladesh Medical Association opposes the idea on patient safety grounds, whereas the Bangladesh Nurses Association is in support with proper training and regulatory mechanisms in place [15].

## Conclusion

The acute shortage of health human resource in Bangladesh retards the efficient functioning of the health systems of the country. The impact of this shortage, which is very unlikely to be met in the foreseeable future, is felt more in the rural areas as the limited supply of physicians tends to concentrate in the urban areas. In the face of limited and sometimes inaccessible formal healthcare, it is the Village Doctors who the villagers turn to in meeting their demand for healthcare. Village Doctors, if properly trained, hold a huge potential in ensuring primary healthcare for the rural people. Formal health systems should find ways to be inclusive in terms of the role of the Village Doctors in providing healthcare to the rural people in Bangladesh.

## Competing interests

The authors declare that they have no competing interests.

## Authors' contributions

SSM conceptualized and drafted the manuscript and also contributed in data analysis and interpretation. MI supervised the study and reviewed the draft. SMAH contributed in collecting, analyzing and interpreting the data. TW contributed in finalizing the manuscript and in literature review. AB was the Principal Investigator of the project under which data used in this paper were collected. He also assisted in data analysis and critically reviewed the manuscript for consistency and significant additions. All the authors read and approved the final manuscript.

## Pre-publication history

The pre-publication history for this paper can be accessed here:

http://www.biomedcentral.com/1472-698X/10/18/prepub
